# Development and content validation of the Telenursing Interaction and Satisfaction Questionnaire (TISQ)

**DOI:** 10.1111/hex.12945

**Published:** 2019-09-12

**Authors:** Marie Mattisson, Christina Johnson, Sussanne Börjeson, Kristofer Årestedt, Malou Lindberg

**Affiliations:** ^1^ Department of Medical and Health Sciences Linköping University Linköping Sweden; ^2^ 1177 Medical Advisory Service and Department of Medical and Health Sciences Linköping University Linköping Sweden; ^3^ Faculty of Health and Life Sciences Linnaeus University Kalmar Sweden; ^4^ The Research Section Kalmar County Council Kalmar Sweden

**Keywords:** communication, content validity, nurse‐patient relations, patient experiences, patient satisfaction, surveys and questionnaires, telenursing

## Abstract

**Background:**

Caller satisfaction with telephone advice nursing (TAN) is generally high, and the interaction is essential. However, a valid questionnaire exploring caller satisfaction in TAN with focus on perceived interaction is lacking.

**Objective:**

To develop and assess content validity and test‐retest reliability of a theoretically anchored questionnaire, the Telenursing Interaction and Satisfaction Questionnaire (TISQ), that explores caller satisfaction in TAN by focusing on perceived interaction between the caller and the telenurse.

**Methods:**

The study was performed in three stages. First, variables relevant for patient satisfaction in health care were identified through a literature search. Variables were then structured according to the Interaction Model of Client Health Behavior (IMCHB), which provided theoretical guidance. Items relevant for a TAN context were developed through consensus discussions. Then, evaluation and refinement were performed through cognitive interviews with callers and expert ratings of the Content Validity Index (CVI). Finally, test‐retest reliability of items was evaluated in a sample of 109 individuals using intraclass correlation coefficients (ICC).

**Results:**

The TISQ consists of 60 items. Twenty items cover perceived interaction in terms of health information, affective support, decisional control and professional/technical competence. Five items cover satisfaction with interaction and five items overall satisfaction. Remaining items reflect singularity of the caller and descriptive items of the call. The TISQ was found to exhibit good content validity, and test‐retest reliability was moderate to good (ICC = 0.39‐0.84).

**Conclusions:**

The items in the TISQ form a comprehensive and theoretically anchored questionnaire with satisfactory content validity and test‐retest reliability.

## 1 INTRODUCTION

The field of telephone advice nursing (TAN) has expanded rapidly in western countries during the past decade,^1^ and for many patients, the interaction with the nurse is the first contact with health care. The easy access to professional advice in health matters is perceived as a reliable asset in daily life.^2^ Research has provided support for its benefits,^1^, ^3^ and the service continues to grow.

In TAN, the interaction between the caller and the telenurse takes place during a relatively short and limited amount of time and is predominantly based on verbal communication. The interaction could further be described as a fundamental base within which the nursing process is accomplished.^4^ In a recent concept analysis within a nursing care context,^4^ it is suggested that nurse‐patient interactions consist of following attributes: an overall aim towards facilitation of health; verbal or non‐verbal exchange; dynamic adaptation; and multi‐dimensionality such as physical, psychological, social or spiritual dimensions. The interaction and its meaning is perceived uniquely by each patient and nurse, and factors influencing the perception include health concerns, knowledge, interpersonal style, setting and expectations, as preferences for how the interaction will proceed.

**Figure 1:**
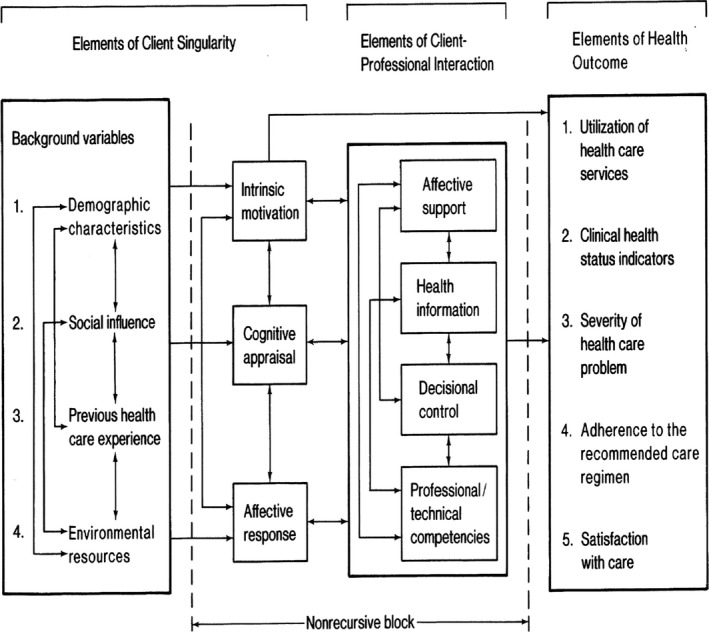
Interaction Model of Client Health Behavior 5

Components of the interaction process and how they relate to outcomes such as patient satisfaction are described in the Interaction Model of Client Health Behavior (IMCHB) by Cox^5^ (Figure 1). The object of this model is to ‘identify and suggest explanatory relationships between client singularity, the client‐provider relationship and subsequent client health‐care behaviour’.^5^ The model is generic for nursing purposes but according to its originator most useful in nursing situations when the client's personal responsibility and control of the health problem is large and the role of the health‐care professional is more of an advisor, teacher or technician.^5^

The IMCHB describes the interaction process as a major influence on health‐care outcomes such as satisfaction. Four components define the content of the interaction process: health information; affective support; decisional control; and professional/technical competence of the nurse. The professional nurse should ideally tailor the interaction with the patient depending on factors relating to the unique client and his or her expressed need for health care (client singularity), also described as a the dynamic qualities of the interaction by Evans.^4^ Thus, the four components of interaction in the IMCHB work towards achieving health outcomes in terms of further use of health‐care services, change in clinical health status, change in severity of the health‐care problem, adherence to recommended care regimen and satisfaction with care.^5^

High patient satisfaction rates have been considered a desired outcome and even a component of quality of care itself.^6^ It is also considered a predictor of future behaviour.^7^ In spite of the relatively large number of studies on patient satisfaction, according to Batbaatar et al^8^ there is still no widely adopted definition of the concept within a health‐care context, and study results trying to detect its determinants within health care are inconclusive and sometimes contradictory. The following is one way the nursing field defines patient satisfaction: ‘[T]he patient's subjective evaluation of the cognitive/emotional response that results from the interaction of the patient's expectations of nursing care and their perception of actual nurse behaviours/characteristics’.^9^ This definition indicates that patient satisfaction with nursing care is a complex combination of factors including expectations and other socio‐psychological factors as well as perceptions of delivered care.

Chow et al^10^ describe patient satisfaction as the result of determinants and components. In this model, determinants refer to patient characteristics such as demographic variables as well as expectations about care. Components refer to different aspects of actual care delivered in terms of affability, ability and availability. Affability refers to interpersonal manners of the medical staff, ability to health‐care professional or technical quality, and availability to accessibility issues. According to a literature review by Batbaatar et al,^11^ interpersonal care quality is the most important factor that influences satisfaction with care.

Since the general shift towards increased patient influences in health care, patient satisfaction has been widely studied and a large number of surveys to measure the trait have been developed. Criticism of these measures includes a lack of conceptualization, low standardization, low reliability and uncertain validity,^12^ which prevent meaningful comparisons between existing satisfaction assessments. Measures of patient satisfaction have been used interchangeably with measures of perceived service quality, a fact criticized by Gill and White,^12^ who call for a separation of the two concepts. In a systematic review by Allemann Iseli et al,^13^ 16 published instruments measuring patient and caller satisfaction with out‐of‐hours services and teleconsultation and triage were examined. A majority of the reviewed instruments showed limitations in methodology and insufficient evaluation. For instance, only a few of the 16 instruments provided detailed information on item generation and content validation methodology,^13^ which reduces possibilities to assess usability in other contexts.

In TAN, reported satisfaction with calls is generally high,^2^, ^14^ but, as described above, the degree of satisfaction is not necessarily a measure of high quality of care. It could, for example, be the result of low expectations and is affected by gender and age, as described by Chow et al^10^ Parallel to this, there is in literature on TAN a documented need for improvements in health‐care quality in terms of telenurses’ communication competence,^15^, ^16^ and it has been suggested that patient satisfaction surveys designed for a TAN context should monitor improvements in telenurses’ communication competence.^17^ To our knowledge, there is no survey available that examines both the perception of and the satisfaction with the different parts of the interaction with the telenurses accompanied by the large number of potential influencing variables presented in the IMCHB. Thus, there is a need for a thoroughly developed questionnaire enabling systematic investigations on interactional matters, how they are perceived by callers and how they correlate to caller satisfaction. For content validity reasons, transparency in the development and validation process of such a questionnaire is needed.^18^ Therefore, the aim of this study was to develop and assess content validity and test‐retest reliability of items of a theoretically anchored questionnaire, the TISQ, that explores caller satisfaction in TAN with focus on perceived interaction between the caller and the telenurse.

## 2 METHODS AND RESULTS

In this study, the person who makes the phone call is referred to as ‘the caller’ and could be either the patient or a person calling on behalf of the patient. All aspects of perceptions and satisfaction in this study refer to the person participating in the interaction with the telenurse, whether or not he or she is the patient.

**Figure 2:**
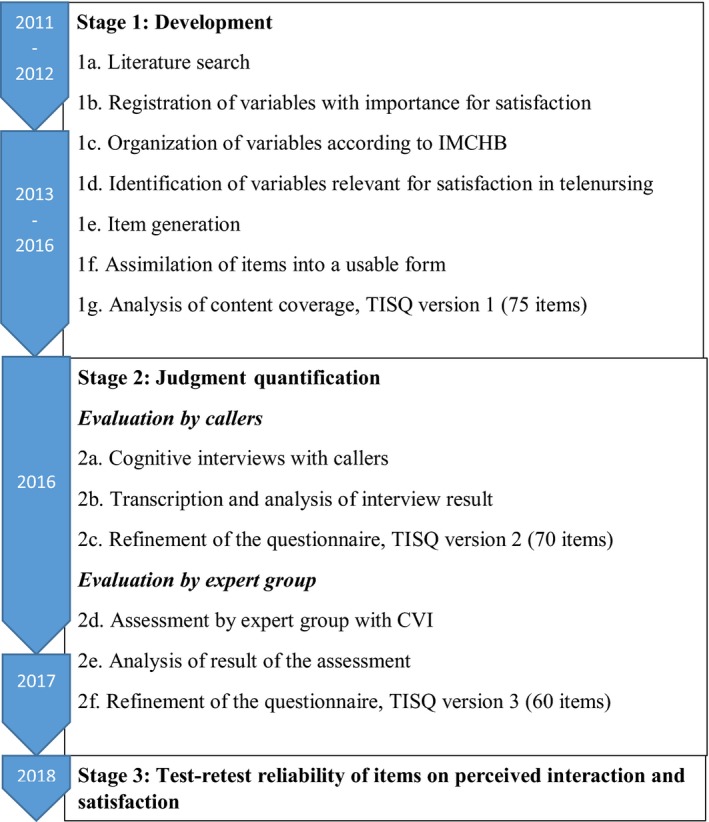
The development process for the Telenursing Interaction and Satisfaction Questionnaire (TISQ)

The process of developing the Telenursing Interaction and Satisfaction Questionnaire (TISQ) was divided into three stages: development and judgement quantification, as suggested by Lynn,^19^ and evaluation of test‐retest reliability.^20^ In the first stage, a literature search was accomplished to identify the domain of satisfaction in TAN. Item generation was performed.^21^ In the second stage, judgement quantification, the process was separated into two phases: cognitive interviews with callers^22^ and evaluation by experts using the Content Validity Index (CVI).^23^, ^24^ The results from cognitive interviews and the CVI guided revisions of the entire questionnaire. In the third stage, test‐retest reliability of items on perceived interaction and satisfaction was evaluated using intraclass correlation coefficients (ICC). The process is illustrated in Figure 2.

### 2.1 Stage 1: development

Identification of the domain (steps 1a‐d; Figure 2), item generation (step 1e; Figure 2) and assimilation of items into a useable form (step 1f; Figure 2) were performed, and content coverage was analysed (step 1g; Figure 2).

#### 2.1.1 Identification of the domain

An initial literature search was conducted in PubMed and Cinahl between 2011 and 2012. The aim was to identify variables of importance for satisfaction in TAN. Due to a limited number of studies on telenursing, the search was broadened to include perceptions of satisfaction with nursing and health care in general as well as to find existing questionnaires measuring satisfaction with nursing care in different settings. The search terms used were telenursing, patient/caller satisfaction, patient/caller perceptions, nursing care and questionnaire. Additional studies and questionnaires were identified by examining reference lists. The search also included questionnaires on patient and/or consumer satisfaction developed and published by Swedish authorities on their official websites. A total of 31 relevant studies and questionnaires were selected to provide a wide perspective of the domain, and 13 of these sources focused specifically on different aspects of telenursing.

All sources were scrutinized in a search for relevant variables. Approximately 300 variables were registered. Variables were then structured according to the headings in the IMCHB. Through consensus discussions in the author group, including expert knowledge in telenursing, nursing research and instrument development, the initial 300 variables were merged into 75 variables, considered relevant for a TAN context and representing all categories in the IMCHB. The domain was therefore identified and defined by the structure of the existing theoretical model, with one exception: the items representing satisfaction were separated into two subcategories that were not present in the IMCHB—overall satisfaction and satisfaction with interaction.

#### 2.1.2 Item generation and assimilation of items into useable form

The next step in the development stage was converting variables into items. Wording was discussed in the author group with respect to interpretability in terms of reading level requirements, ambiguity, double‐barrelled wording, jargon, value‐laden words, and positive and negative wording. Options for response alternatives were discussed until a consensus was reached. Effort was put into ensuring a possible response alternative for every respondent and situation. Items were then assembled into a usable form.

Content coverage^21^ was checked according to the headings in IMCHB. Every subheading of client singularity (background and dynamic variables) and client‐profession interaction was represented by at least one item in the questionnaire. Health outcome was represented by items on satisfaction, and other outcome variables were excluded. Content coverage was also checked in relation to a previously developed telenursing communication self‐assessment tool^25^ in order to ensure that aspects of nursing communication competence and phases of the nursing process were adequately covered. This first version of the TISQ consisted of 75 items.

### 2.2 Stage 2: judgement quantification

Content validity and understandability were evaluated from both caller and expert perspectives. First, cognitive interviews with callers were performed (steps 2a‐c; Figure 2). Then, content validity was evaluated from a professional point of view using the Content Validity Index (CVI) (steps 2d‐f; Figure 2). Revision of the questionnaire was guided by the results from both methods.

#### 2.2.1 Evaluation by callers—cognitive interviews

Cognitive interviews according to verbal probing technique were conducted individually with six callers. Nurses at the Swedish National Telephone Advice Nursing service (1177) in the region of Östergötland were asked to identify and invite a purposeful selection of callers who presented diversity in terms of sex, age of the patient, time of call, estimated language skills, satisfaction, estimated complexity of the problem and estimated degree of anxiety. The sample consisted of three women and three men, all fluent in Swedish, age ranging from 25 to 75 years. In addition, the sample fulfilled the above criteria for estimated anxiety, satisfaction and complexity of the problem. The callers were free to choose the location of the interview, either at home or in a neutral location. The callers were presented with the questionnaire and instructed to read and answer every question aloud. Callers were encouraged to think out loud about their interpretation and acceptance of items and response options, and about the cognitive process that took place while answering the questions. Open‐ended verbal probes prepared before the interviews encouraged callers to expand their answers. All interviews were recorded and transcribed verbatim. The median length of the cognitive interviews was 56 minutes (45‐85 minutes). The median time elapsed from the actual call to the interview was 15 days (4‐28 days).

Transcriptions were used to support revisions to improve the questionnaire. Miscomprehensions of wording and entirety were revealed, as were problems with memory recall, motivation and response processes. Further refinement of items, response options, headlines and instruction texts were discussed within the author group, and revisions were made with respect to the IMCHB. In all, six items were deleted due to perceived similarities and irrelevance: one on client singularity; one on expectations on support; one on overall satisfaction; and three on perceived affective support. One item on estimated number of previous calls to this service was added, and 35 items were reworded. The order of the questions was revised with respect to caller comments. After this refinement, the TISQ consisted of 70 items.

#### 2.2.2 Evaluation by experts—content validity index

Further evaluation of the TISQ was performed using the Content Validity Index (CVI). The goal was to include a carefully selected and purposeful sample of expertise within communication in health care in general, TAN, instrument development, evaluations of health care and clinically active telenurses. Sixteen experts were thus invited to participate individually in the content validity process: 9 researchers in the fields of telenursing, quality of care, communication in nursing and instrument development; five clinically active and/or experienced telenurses; and two people with professional experience in human sciences and evaluations of health‐care quality.

Information about the background and purpose of the questionnaire was posted along with instructions on how to complete the attached evaluation form. Specific instructions were given to judge the clarity of items and the comprehensiveness of the entire questionnaire and to suggest any additional items. The experts were instructed to rate the relevance of each item from 1 to 4, where 1 indicated ‘not relevant’ and 4 indicated ‘highly relevant’. Experts were also encouraged to share comments concerning the relevance and wording of items and response options, suggestions for revision, number and ordering of items, instruction texts, missing items, headlines and layout. Finally, the experts were asked to rate the overall relevance of the entire questionnaire in the same manner, from 1 (“not relevant”) to 4 (“highly relevant”).

Responses were received from 13 experts: four researchers representing all research fields specified above; three clinically active and/or experienced telenurses; and two professionals within the fields of human science and evaluations of health‐care quality. Another three experts chose to answer anonymously, and one expert chose to comment on questionnaire construction issues and not the CVI‐rating.

Item CVIs (I‐CVIs) were computed by dividing the number of experts rating 3 (“relevant”) or 4 (“highly relevant”) by the total number of experts completing the rating. Items with I‐CVIs of 0.78 or lower were considered to need revision or to be deleted.^23^ I‐CVIs ranged from 0.64 to 1.0. Three items had I‐CVIs of 0.78 or lower. Two of these—one concerning social influence and one on total number of previous contacts with health care—were deleted. The third item with I‐CVI of 0.70 concerned expectations on decisional control. In the cognitive interviews, this item yielded great variance in comments depending on differences in expectations on the role of the telenurse. A few callers viewed the telenurse as the self‐evident expert with full mandate to make decisions without caller involvement, while others were more prone to participate in the decision making, depending on health status and own level of knowledge about the problem when calling. With this in mind and with support from theory, the item was considered valuable for satisfaction and retained after revision in spite of unacceptable I‐CVI. Eight items with acceptable I‐CVIs—four on affective support, three on health information and one on decisional control—were deleted due to expert comments on similarities between items. For example, one item on whether the nurse was honest and sincere was deleted since it resembled the item on confidence in the telenurse. All written expert comments were considered, including in cases of acceptable I‐CVIs. I‐CVI values are presented in Table 1. The CVI of the entire questionnaire based on the experts’ ratings was 0.92. In addition, scale CVI Average (S‐CVI/Ave) was calculated as the mean of all I‐CVIs. The S‐CVI/Ave of the TISQ was also 0.92, which is above the acceptable level of 0.9.^23^ No further evaluation of CVI was performed after the revision.

**Table 1:** Items included in the TISQ, content validity and test‐retest reliability. Descriptive items excluded. Back‐forward translation has not been carried out

IMCHB heading	Items in the TISQ	Item CVI^a^	ICC^b^	95% CI for ICC
Client singularity—background variables				
Demographic characteristics	(sex, age, native country, level of education, daily occupation, household economy, health status)	‐		
Social influence	‐	‐		
Previous health‐care experience	Did you expect to get what you wanted?	0.82		
	When you called, did you expect the nurse to take your problem seriously?	0.83		
	When you called, did you expect the nurse to listen carefully to you?	0.91		
	When you called, did you expect the nurse to show you respect?	0.9		
	When you called, did you expect the nurse to treat you well?	0.9		
	When you called, did you expect the nurse to provide accurate and useful information?	0.91		
	When you called, did you expect the nurse to give you the opportunity to influence the result of the call?	0.7		
	When you called, did you expect the nurse to have enough competence to deal with your health problem?	0.91		
	Except for this call, how many times have you previously contacted XXX?	0.91		
	Overall, how satisfied are you with your previous calls to XXX?	0.91		
	Overall, how satisfied are you with your previous contact with health care?	0.82		
Environmental resources	Have you made use of any sources other than XXX for advice and help with this particular health problem?	0.82		
Client singularity—dynamic variables				
Intrinsic motivation	When you called, what did you most want to happen as a result of the call?	1.0		
	How strong was this desire (XX) when calling?	0.92		
Cognitive appraisal	What was your perceived urgency for an answer or a solution to your health problem?	1.0		
Affective response	How worried were you when calling?	1.0		
Client‐profession interaction				
Health information	When you called, did you perceive that you were given the opportunity to ask all your questions?	1.0	0.52	0.36‐0.65
	When you called, did you perceive that you received answers to all your current questions at the time?	1.0	0.72	0.62‐0.80
	When you called, did you perceive that the nurse provided you with information on the future potential development of the health problem?	1.0	0.55	0.39‐0.67
	When you called, did you perceive that you got information about what you should do next?	1.0	0.68	0.55‐0.77
	When you called, did you perceive that you had understood the advice/information when ending the call?	1.0	0.52	0.36‐0.65
	When you called, did you perceive that you received advice and information adapted to your needs and conditions at the time?	0.92	0.75	0.65‐0.83
	When you called, did you perceive that you were informed about where to find additional information?	1.0	0.61	0.46‐0.72
Affective support	When you called, did you perceive that you felt confidence in the nurse you talked to?	1.0	0.67	0.54‐0.76
	When you called, did you perceive that the nurse listened attentively?	1.0	0.71	0.61‐0.80
	When you called, did you perceive that the nurse understood what you wanted?	0.92	0.51	0.36‐0.64
	When you called, did you perceive that the nurse showed empathy?	0.83	0.73	0.63‐0.81
	When you called, did you perceive that the nurse was friendly?	1.0	0.71	0.59‐0.79
	When you called, did you perceive that the nurse was calm and instilled a sense of security?	0.83	0.73	0.62‐0.81
	When you called, did you perceive that the nurse showed an interest in your understanding of the health problem?	0.83	0.58	0.44‐0.70
Decisional control	When you called, did you perceive that you were given opportunities to discuss alternative solutions to the health problem?	1.0	0.72	0.60‐0.80
	When you called, did you perceive that you and the nurse agreed on how to deal with your health problem?	1.0	0.73	0.63‐0.81
Professional‐technical competencies	When you called, did you perceive that the nurse had enough competence to deal with your health problem?	0.92	0.78	0.69‐0.84
	When you called, did you perceive that the nurse asked relevant questions about your health problem?	1.0	0.67	0.55‐0.77
	When you called, did you perceive that the nurse was thorough in her work?	0.92	0.68	0.56‐0.77
	When you called, did you perceive that the nurse was skilled in leading the conversation forward?	0.91	0.79	0.71‐0.86
Health outcome—satisfaction with care				
Satisfaction (with interaction)	Overall, how satisfied were you with the advice and information you were given?	1.0	0.83	0.77‐0.88
	Overall, how satisfied were you with the competence of the nurse?	1.0	0.84	0.78‐0.89
	Overall, how satisfied were you with the nurse's ability to support you affectively?	1.0	0.58	0.44‐0.69
	Overall, how satisfied were you with how the nurse treated you?	1.0	0.67	0.54‐0.76
	Overall, how satisfied were you with the possibility to influence the result of the call?	1.0	0.51	0.36‐0.64
Satisfaction (overall)	How satisfied were you with the result of the call according to question number XX?	1.0	0.72	0.62‐0.80
	Overall, did you experience that your expectations were met?	1.0	0.79	0.70‐0.85
	If a new health problem were to occur in the future, would you then wish to speak to the same nurse again?	0.91	0.39	0.22‐0.54
	Finally, how satisfied were you as a whole with the current call to XX?	0.91	0.73	0.62‐0.81
	What would make you more satisfied with this call to XX?	1.0	‐	‐

Abbreviations: IMCHB, Interaction Model of Client Health Behavior by Cox.^5^ TISQ, Telenursing Interaction and Satisfaction Questionnaire.

a Item Content Validity Index refer to expert ratings before final revision of wording.

b Intraclass correlation coefficient, two‐way mixed‐effects model, absolute agreement (N = 109).

Information letters to respondents and instruction texts were also revised due to expert comments as were headlines, response options and sequencing of items. All revisions were made after reaching consensus within the author group. Also, no revisions were implemented before checking in accordance with the IMCHB and results from previous stages in the development process.

### 2.3 Stage 3: test‐retest reliability of items on perceived interaction and satisfaction

For evaluation of test‐retest reliability of items on perceived interaction and satisfaction (stage 3; Figure 2), a consecutive sampling procedure was conducted from the Swedish National Telephone Advice Nursing service (1177) for 5 weeks in 2017. At the beginning of every call, an automatic response message informed and invited callers about the study and invited them to participate. Inclusion criteria were age of 18 years or older, calling on behalf of own health problem, and cognitively and linguistically capable to communicate in Swedish. Questionnaires were posted 2‐5 days after the registered call to recipients who accepted participation in the study. In addition to the questions in the TISQ, callers were asked if they wanted to answer the questionnaire twice for test‐retest purposes, and 168 individuals accepted this. The instruction was to complete questionnaire number two within 1 or 2 weeks, but answers were collected up to 30 days after the first questionnaire was completed. In total, 109 retest questionnaires were returned.

Intraclass correlation coefficient (ICC, two‐way mixed and absolute agreement) was used to evaluate test‐retest reliability of the 20 items on perceived interaction and nine items on satisfaction (Table 1). The following criteria were used^20^ to support test‐retest reliability: <0.5 poor; 0.5‐0.75 moderate; 0.75‐0.9 good; and >0.9 excellent.

A majority of the items (n = 22) showed moderate reliability (ICC = 0.51‐0.73). Six items showed good reliability (ICC = 0.75‐0.84), and one item demonstrated poor reliability (ICC = 0.39) but was kept in this version of the questionnaire (Table 1).

### 2.4 The final version of the Telenursing Interaction and Satisfaction Questionnaire (TISQ)

After this revision process, the TISQ consisted of 60 items: 23 on client singularity, 20 on perceived interaction, ten on satisfaction and an additional seven items on the description of the call.

The items in the TISQ are sorted into four separate sections. The first section includes items on the caller's appraisal of the situation and expectations prior to the call. The second section contains items about the caller's perceived interaction with the nurse and is divided into four subgroups according to the IMCHB: affective support, health information, decisional control and professional/technical competence. Satisfaction item(s) directly follow each of the four subgroups on perceived interaction. The third section in the TISQ consists of items covering overall patient satisfaction with the call. The fourth section includes descriptive items about the specific call (result of the call, timing, if the caller called on behalf of someone else, waiting time, preventive counselling and whether the call was carried out in Swedish or another language) and the caller's demography (sex, age, education, daily occupation, household economy, native tongue and general health condition).

## 3 DISCUSSION

This study describes the thorough process of developing a theoretically anchored content valid questionnaire exploring callers’ perceptions of the interaction with the telenurse and caller satisfaction. This is, to our knowledge, the first comprehensive questionnaire focusing on caller satisfaction and interaction between the caller and the telenurse. It derives from an identification of the domain and is structured according to the IMCHB, a nursing model that recognizes the interaction process as vital for health outcomes such as satisfaction.

The main purpose of the TISQ is to enable systematic investigations on interactional matters, how callers perceive these matters and how these matters correlate to caller satisfaction. Therefore, all potential influencing variables must be represented. The TISQ will not provide multi‐item scales for measurement of satisfaction with calls, but merely provide a set of content valid items covering the complexity of patient satisfaction in TAN. Therefore, traditional psychometric analyses are not appropriate for evaluation at this stage. Terwee et al^18^ state that content validity is the most important measurement property of patient‐reported outcome measures. According to the COSMIN checklist,^18^ criteria regarding item relevance, appropriateness of response options and recall period, comprehensiveness and comprehensibility must be fulfilled to achieve good content validity and that the target population as well as appropriate expertise should be involved in this process. A majority of studies reporting on satisfaction instruments do not provide a detailed record of their development including theoretical underpinnings and conceptualization of the trait.^13^

Content validity should always be assessed in relation to context. The TISQ exhibits good content validity in its intended area of use: telephone calls concerning all kinds of health matters from the entire population to the nurse‐led Swedish National Medical Health Advisory Service (1177). For example, response options related to levels of care are adjusted according to the facilities in Sweden and may need adjustment before valid use in other contexts. However, the theoretical foundation of the TISQ derives from international literature. This would support external validity in other TAN contexts where potential client responsibility and control of the health problem are large.

The literature search in this study was accomplished without preconceptions. It was guided by research questions on what callers actually perceive when calling for advice and descriptions of satisfaction in nursing literature. It could be argued that the search for relevant literature should have been continuous during the entire development process; however, to our knowledge, since the initial literature search, there have been few contributions to the telenursing field that would have changed the content of the TISQ. This fact was further confirmed when comparing the items in the TISQ with the results of reviews published after completion of the literature search.^11^, ^13^, ^26^

Existing theory on determinants to patient satisfaction is, as described, complex and somewhat diverging.^11^ The IMCHB by Cox^5^ was chosen to provide a theoretical and sufficiently complex foundation for the content of the TISQ that at the same time provided guidance to identification of domains. Research studies have suggested the IMCHB to be a useful and comprehensive guide in nursing research.^27^, ^28^ In addition, the focus on interactional matters in the IMCHB is well adapted for the purposes of the TISQ. When using the model, it is recommended to focus on one or two of the outcomes,^27^ which is the case in the TISQ, where all outcomes except satisfaction have been excluded.

One of the methodologies for judgement of the questionnaire—cognitive interviews with callers—added insight in addressing concerns experienced by the callers. This perspective is valuable for content validity reasons^18^ but is also of importance for the future respondents’ motivation to complete the questionnaire.^29^ The verbal probing technique applied in the study gave insight to some beforehand important issues. For example, the interviews supported callers’ ability to distinguish between desired and expected care and revealed divergent interpretations of key terms such as “severity”, “anxiety” and “result of the call”. These are everyday words that the callers most likely would not have reflected on otherwise.

Professional expert input contributed to the validation process through the method of CVI. This method is well documented and widespread in science.^23^ It is recommended due to its ease of computation, understandability, focus on agreement of relevance, and provision of both item and questionnaire information. The procedure of letting experts share comments, especially on items with low‐rated relevance, was helpful in the revision process as it provided explanations for low ratings and suggestions for revision.

The final version of the TISQ includes one item with I‐CVI of 0.7 concerning caller expectations of influencing the result of the call. The issue of expectations as a predictor of satisfaction in TAN has support in theory[11, 14, 17, 30] and is pointed out as being essential in the definition of patient satisfaction by Eriksen.^9^ Therefore, this item was kept unrevised in spite of low I‐CVI. In the IMCHB, expectations are integrated in client singularity, but this is not represented as one explicit factor. When expectations are not met, the telenurse's communication competence seems to have an important impact on satisfaction.^17^ Reasons for met or unmet expectations could derive from the patient's perspective but could also be a result of telenurses’ diverging understanding of professional responsibilities.^31^ If a telenurse mainly focuses on optimising availability and ‘gate‐keeping’, it is likely that potentially more time‐consuming dialogues such as affective supporting and health‐promoting dialogues will be avoided and vice versa. Satisfaction or dissatisfaction could occur either way, depending on the expectations of the caller, which is why exploring the patients’ expectations is important for satisfaction optimization. According to Batbaatar et al,^8^ there is no globally accepted knowledge about how unmet expectations affect patient satisfaction, and further research is recommended. The TISQ may contribute more knowledge about how unmet expectations affect satisfaction in telephone advice nursing, and thus future studies may evaluate the usefulness of this specific item.

After completion of the cognitive interviews, the TISQ was revised and the professional experts were thus presented with a second version of the questionnaire. The final version after revision due to expert evaluation might have been slightly different if the evaluations had been performed in the opposite order. According to the COSMIN checklist, cognitive interviews should be performed of the final version of any patient‐reported outcome measure. However, in this study, all revisions at all stages were made with respect to results from previous stages in the process and theoretical findings.

Test‐retest reliability of interaction and satisfaction items was acceptable for all items except one item with poor reliability. One reason for the relatively moderate levels could be that items are unclear or badly worded. However, this picture did not emerge in the cognitive interviews, where items were found to be clear and easy to understand. More likely, the constructs in focus—that is perceived interaction and satisfaction—are not stable and change over time. According to the instructions to respondents, the second questionnaire was to be completed and returned within 1‐2 weeks from the first assessment, but many retest questionnaires were delayed and collected up to 4 weeks after the first assessment. According to Jackson et al,^32^ low correlations between immediate and follow‐up satisfaction measures may be explained by the fact that immediate assessments are more likely to be influenced by the actual meeting with the clinician and later assessments by improvement of symptoms. This highlights the importance of early distribution timing of the TISQ. Because no data on time elapsed from the actual call to measuring point are collected in the TISQ, the importance of this cannot be assessed in this study.

In measurement of patient satisfaction, it is a well‐known fact that satisfaction rates tend to be high^14^, ^33^ and dissatisfaction only emerges in situations where there are obvious reasons. In an attempt to minimize these routine high satisfaction ratings, fairly detailed items on perceived interaction are in the TISQ directly followed by satisfaction rating(s) on that specific interaction element. The purpose of this approach was to guide respondents into distinguishing between perceived quality of health care and satisfaction^12^ and to elicit nuances of satisfaction if possible. As discussed in a review by Sitzia and Wood,^33^ item construction in terms of general or detailed items may affect the result of satisfaction reports. There is a risk that respondents will assume questions are basically the same and maintain consistency in their answers, not really reading the questions. Comments on the relatively large number of items were collected from both callers and the group of experts. Nonetheless, callers participating in the cognitive interviews appreciated the opportunity to share a fair picture of the call, which has been described in theory.^29^ The choice of a relatively large number of items on perceptions is further supported by Gill and White,^12^ and therefore, no further deletion of items was performed at this stage. Parts of the TISQ will be further evaluated in terms of psychometric properties that might support further reduction of items.

## 4 CONCLUSION

This study describes the thorough process of developing and assessing content validity of the Telenursing Interaction and Satisfaction Questionnaire (TISQ). The TISQ will enable further understanding about the relationships between callers’ perceptions of the interaction process with the telenurse and satisfaction with calls. With better knowledge about this, communication improvement and education in telenursing can be tailored to enhance caller satisfaction. It may also contribute knowledge about how client singularity, including both dynamic and non‐dynamic variables, affects satisfaction with telenursing. Knowledge in these areas enables evidence‐based development of communication education and training programmes in the clinical practice of TAN.

## CONFLICT OF INTEREST

The authors declare that there is no conflict of interest.

## ETHICAL APPROVAL

This study was performed according to the Principles of the Declaration of Helsinki (World Medical Association 2013). All involved callers and experts were given information about the study and were informed that their participation was voluntary. Both the panel of experts and the callers were guaranteed that all data would be treated confidentially. No information reported in this paper can be linked to any individual. The study has been approved by the regional ethical review board in Linköping, Sweden (No. 2015/298‐31).

## Data Availability

The data that support the findings of this study are available from the corresponding author upon reasonable request.

## References

[1] Souza‐Junior VD , Mendes I , Mazzo A , Godoy S . Application of telenursing in nursing practice: an integrative literature review. Appl Nurs Res. 2016;29(1):254‐260. 26856523 10.1016/j.apnr.2015.05.005

[2] Ström M , Marklund B , Hildingh C . Callers' perceptions of receiving advice via a medical care help line. Scand J Caring Sci. 2009;23(4):682‐690. 19807883 10.1111/j.1471-6712.2008.00661.x

[3] Marklund B , Ström M , Mänson J , Borgquist L , Baigi A , Fridlund B . Computer‐supported telephone nurse triage: an evaluation of medical quality and costs. J Nurs Manag. 2007;15(2):180‐187. 17352701 10.1111/j.1365-2834.2007.00659.x

[4] Evans EC . Exploring the nuances of nurse‐patient interaction through concept analysis: impact on patient satisfaction. Nurs Sci Quart. 2015;29(1):62‐70. 10.1177/0894318415614904 26660778

[5] Cox CL . An interaction model of client health behavior: theoretical prescription for nursing. Adv Nurs Sci. 1982;5:41‐56. 10.1097/00012272-198210000-00007 6817699

[6] Donabedian A . Explorations in Quality Assessment and Monitoring. Ann Arbor, MI: Health Administration Press; 1982.

[7] Pascoe GC . Patient satisfaction in primary health care: a literature review and analysis. Eval Prog Plan. 1983;6(3):185‐210. 10.1016/0149-7189(83)90002-2 10299618

[8] Batbaatar E , Dorjdagva J , Luvsannyam A , Amenta P . Conceptualisation of patient satisfaction: a systematic narrative literature review. Perspect Public Health. 2015;135(5):243‐250. 26187638 10.1177/1757913915594196

[9] Eriksen LR . Patient satisfaction with nursing care: concept clarification. J Nurs Meas. 1995;3(1):59‐76. 7493189

[10] Chow A , Mayer EK , Darzi AW , Athanasiou T . Patient‐reported outcome measures: the importance of patient satisfaction in surgery. Surgery. 2009;146(3):435‐443. 19715800 10.1016/j.surg.2009.03.019

[11] Batbaatar E , Dorjdagva J , Luvsannyam A , Savino MM , Amenta P . Determinants of patient satisfaction: a systematic review. Perspect Public Health. 2017;137(2):89‐101. 27004489 10.1177/1757913916634136

[12] Gill L , White L . A Critical Review of Patient Satisfaction. Leadership in Health Services. Great Britain: Emerald Group Publishing Limited; 2009:8–19.

[13] Allemann Iseli M , Kunz R , Blozik E . Instruments to assess patient satisfaction after teleconsultation and triage: a systematic review. Patient Prefer Adherence. 2014;8:893‐907. 25028538 10.2147/PPA.S56160 PMC4077851

[14] Lake R , Georgiou A , Li J , et al. The quality, safety and governance of telephone triage and advice services – an overview of evidence from systematic reviews. BMC Health Serv Res. 2017;17(1):614. 28854916 10.1186/s12913-017-2564-x PMC5577663

[15] Ernesater A , Engstrom M , Winblad U , Holmstrom IK . A comparison of calls subjected to a malpractice claim versus 'normal calls' within the Swedish healthcare direct: a case‐control study. BMJ Open. 2014;4(10):e005961. 10.1136/bmjopen-2014-005961 PMC4187455 25280808

[16] Ernesater A , Engstrom M , Winblad U , Rahmqvist M , Holmstrom IK . Telephone nurses' communication and response to callers' concern–a mixed methods study. Appl Nurs Res. 2016;29:116‐121. 26856500 10.1016/j.apnr.2015.04.012

[17] Rahmqvist M , Ernesater A , Holmstrom I . Triage and patient satisfaction among callers in Swedish computer‐supported telephone advice nursing. J Telemed Telecare. 2011;17(7):397‐402. 21983224 10.1258/jtt.2011.110213

[18] Terwee CB , Prinsen C , Chiarotto A , et al. COSMIN methodology for evaluating the content validity of patient‐reported outcome measures: a Delphi study. Qual Life Res. 2018;27(5):1159‐1170. 29550964 10.1007/s11136-018-1829-0 PMC5891557

[19] Lynn MR . Determination and quantification of content validity. Nurs Res. 1986;35(6):382‐385. 3640358

[20] Koo TK , Li MY . A guideline of selecting and reporting intraclass correlation coefficients for reliability research. J Chiropr Med. 2016;15(2):155‐163. 27330520 10.1016/j.jcm.2016.02.012 PMC4913118

[21] Streiner DL , Norman GR , Cairney J . Health Measurement Scales: A Practical Guide to Their Development and Use, 5th edn Oxford: Oxford University Press, Cop.; 2015.

[22] Cognitive W . Interviewing ‐ A “How To” Guide. 1999 https://www.chime.ucla.edu/publications/docs/cognitive%20interviewing%20guide.pdf

[23] Polit B , Owen SV . Is the CVI an acceptable indicator of content validity? Appraisal and recommendations. Res Nurs Health. 2007;30(4):459‐467. 17654487 10.1002/nur.20199

[24] Grant JS , Davis LL . Focus on quantitative methods. Selection and use of content experts for instrument development. Res Nurs Health. 1997;20(3):269‐274. 9179180 10.1002/(sici)1098-240x(199706)20:3<269::aid-nur9>3.0.co;2-g

[25] Johnson C , Wilhelmsson S , Börjeson S , Lindberg M . Improvement of communication and interpersonal competence in telenursing ‐ development of a self‐assessment tool. J Clin Nurs. 2015;24(11/12):1489‐1501. 25393698 10.1111/jocn.12705

[26] Kaminsky RM , Björkman A , Holmström IK . Telephone nursing in Sweden: a narrative literature review. Nurs Health Sci. 2017;19(3):278‐286. 28618087 10.1111/nhs.12349

[27] Carter KF , Kulbok PA . Evaluation of the interaction model of client health behavior through the first decade of research. Adv Nurs Sci. 1995;18(1):62‐73. 10.1097/00012272-199509000-00007 7486893

[28] Wagner D , Bear M . Patient satisfaction with nursing care: a concept analysis within a nursing framework. J Adv Nurs. 2009;65(3):692‐701. 19016924 10.1111/j.1365-2648.2008.04866.x

[29] Wenemark M . The respondent's perspective in health‐related surveys [Doctoral thesis, comprehensive summary]. Linköping University Electronic Press; 2010.

[30] Moscato SR , Valanis B , Gullion CM , Tanner C , Shapiro SE , Izumi S . Predictors of patient satisfaction with telephone nursing services. Clin Nurs Res. 2007;16(2):119‐137. 17452431 10.1177/1054773806298507

[31] Kaminsky E , Rosenqvist U , Holmström I . Telenurses' understanding of work: detective or educator? J Adv Nurs. 2009;65(2):382‐390. 19040692 10.1111/j.1365-2648.2008.04877.x

[32] Jackson JL , Chamberlin J , Kroenke K . Predictors of patient satisfaction. Soc Sci Med. 2001;52(4):609‐620. 11206657 10.1016/s0277-9536(00)00164-7

[33] Sitzia J , Wood N . Patient satisfaction: a review of issues and concepts. Soc Sci Med. 1997;45(12):1829‐1843. 9447632 10.1016/s0277-9536(97)00128-7

